# Obesity and height in children and adolescents with acute lymphoblastic leukemia and its future management

**DOI:** 10.18632/oncotarget.26653

**Published:** 2019-02-08

**Authors:** Emily K. Browne, Hiroto Inaba

**Affiliations:** Department of Oncology, St. Jude Children's Research Hospital, Memphis, TN, USA; Department of Pediatrics, University of Tennessee Health Science Center, Memphis, TN, USA

**Keywords:** children, acute lymphoblastic leukemia, obesity, short stature

**Figure 1:**
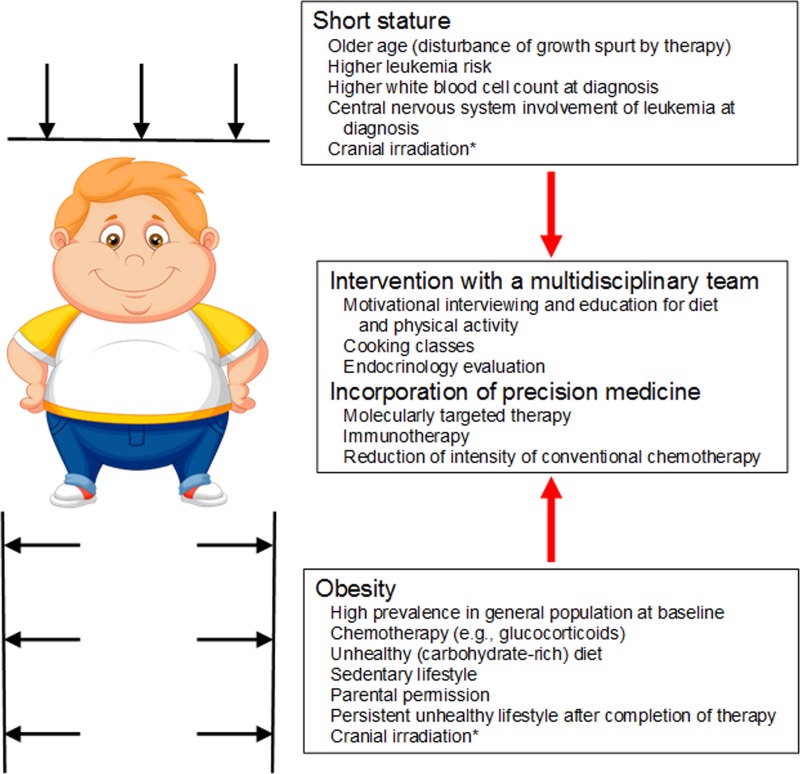
Obesity and short stature in patients with acute lymphoblastic leukemia and survivors *Cranial irradiation is less commonly used in the contemporary treatment regimen for acute lymphoblastic leukemia.

The survival rate for children and adolescents with acute lymphoblastic leukemia (ALL) has improved to more than 90% in resource-rich countries as a result of improved risk-directed treatment and supportive care [1, 2]. However, survivors of ALL often exhibit adverse effects of their treatment, such as an increased risk of obesity and short stature [3] (Figure 1). Obesity can result in substantial physical and psychosocial morbidity, such as infections, hypertension, and hyperglycemia during therapy, as well as metabolic syndrome later in life [4]. Short stature is a well-known complication of ALL treatment, especially when the treatment regimen includes cranial irradiation to control central nervous system (CNS) disease [5]. Recently, we evaluated the longitudinal changes (from diagnosis to 5 years off therapy) in body mass index (BMI), which is a proxy measure of body composition, and height in 372 children and adolescents (aged 2 to 18 years) with ALL who were treated without cranial irradiation on a contemporary treatment regimen [3].

For the BMI analysis, approximately a quarter of the patients were already overweight or obese at diagnosis, reflecting the increased prevalence of obesity in recent pediatric populations [6], but that proportion increased to roughly half of the patients by the time they had been off therapy for 5 years [3]. Importantly, the weight gain started during remission-induction therapy (within 6 weeks after diagnosis), when glucocorticoid is given, and it was further exacerbated after completion of therapy, regardless of the patients' clinical characteristics, such as age, race, sex, or treatment risk. Glucocorticoid stimulates the appetite and energy intake, increases cellular lipid accumulation, and induces insulin resistance [7]. Calorie intake can be unhealthy as a result of a carbohydrate-rich diet, and many patients have a sedentary lifestyle due to chemotherapy-related fatigue and parental permission [8]. Such behavior can persist beyond the completion of ALL therapy.

Height Z-scores decline during treatment and improve after its completion. The height growth in our patients was apparently better than that seen in a historical cohort that received cranial irradiation, but height Z-scores may not return to the levels noted at diagnosis, even in patients treated with intrathecal chemotherapy only. In our study, age ≥10 years at diagnosis, standard/high-risk status, white blood cell (WBC) count ≥ 50 × 10^9^/L at diagnosis, and positive CNS disease were risk factors for decreased height Z-scores, as compared with age 2–9.9 years, low-risk status, WBC count <50 × 10^9^/L, and negative CNS disease, respectively [3]. The growth spurt in adolescents could be affected by chemotherapy, which is more intensive for patients in higher-risk categories and for those with higher WBC counts at diagnosis. This could result in these patients having a lower final height when compared with patients treated at a younger age, although a longer follow-up is needed for the latter population. The frequent intrathecal chemotherapy given to patients with CNS disease at diagnosis and the CNS disease itself may have direct effects on linear growth.

To address this risk of weight gain and short stature, early interventions involving a multidisciplinary team of oncologists, nurses, dietitians, physical therapists, psychologists, and endocrinologists should be implemented, preferably starting during induction therapy. These interventions can include motivational interviewing and parent and guardian education about proper diet (e.g., cooking classes) and physical activity, encompassing reducing the patient's carbohydrate intake and instituting a baseline activity level. For patients with significant height issues, endocrinology evaluation, including screening for growth hormone deficiency, should be considered.

With the advent of whole-genome analysis of germline and leukemia samples, as well as the emergence of immunotherapy, the treatment of ALL will shift further toward a precision medicine approach by incorporating leukemia cell–directed treatment [1, 2]. The use of tyrosine kinase inhibitors (e.g., imatinib, dasatinib, nilotinib, and ponatinib) significantly improved the outcomes for patients with Philadelphia chromosome (*BCR-ABL1*)-positive ALL [9]. The indications for such molecularly targeted agents will expand with the further identification of driving mutations in ALL, such as those causing Philadelphia chromosome‒like (*BCR-ABL1*‒like) ALL and other lesions. For immunotherapy in patients with B-ALL, targeting B cell–specific surface molecules such as CD19 and CD22 with antibodies (e.g., blinatumomab and inotuzumab) has significantly improved the survival rates in adults by comparison with those obtained with conventional chemotherapeutic agents [10]. Chimeric antigen receptor (CAR) T-cell therapy has brought about long-term survival in patients with B-ALL who were previously considered incurable. Such new therapeutic options can decrease the intensity of frontline chemotherapy regimens including glucocorticoids and reduce the acute and long-term adverse effects, which are not limited to obesity and short stature, thus leading to a better quality of cure and a better quality of life for survivors.
